# A Case of Diprosopus: Perinatal Counseling and Management

**DOI:** 10.1155/2014/279815

**Published:** 2014-08-31

**Authors:** Kimberly M. Thornton, Timothy Bennett, Vivekanand Singh, Neil Mardis, Jennifer Linebarger, Howard Kilbride, Kristin Voos

**Affiliations:** ^1^Division of Neonatology, Children's Mercy Hospital, 2401 Gillham Road, Kansas City, MO 64108, USA; ^2^University of Missouri-Kansas City School of Medicine, 2411 Holmes Road, Kansas City, MO 64108, USA; ^3^Department of Obstetrics and Gynecology, Elizabeth J. Ferrell Fetal Health Center, Children's Mercy Hospital, 2401 Gillham Road, Kansas City, MO 64108, USA; ^4^Department of Pathology, Children's Mercy Hospital, 2401 Gillham Road, Kansas City, MO 64108, USA; ^5^Department of Radiology, Children's Mercy Hospital, 2401 Gillham Road, Kansas City, MO 64108, USA; ^6^Department of Pediatrics, Palliative Care Center, Children's Mercy Hospital, 2401 Gillham Road, Kansas City, MO 64108, USA

## Abstract

Diprosopus is a rare congenital malformation associated with high mortality. Here, we describe a patient with diprosopus, multiple life-threatening anomalies, and genetic mutations. Prenatal diagnosis and counseling made a beneficial impact on the family and medical providers in the care of this case.

## 1. Introduction 

Advances in perinatal imaging and diagnostic tools often allow for recognition of complex, rare, and even life-threatening congenital malformations prior to birth. Prenatal diagnosis of these conditions provides time for earlier counseling and planning for perinatal management options. Guiding a family through this process can be difficult for the medical team, but is an attempt to improve the overall outcome and experience for everyone involved. We present a case of diprosopus associated with multiple congenital malformations which were prenatally diagnosed. The parents received extensive multidisciplinary, prenatal counseling allowing both the family and the medical providers to be well prepared for the birth and postnatal management.

## 2. Case 

**Figure 1:**
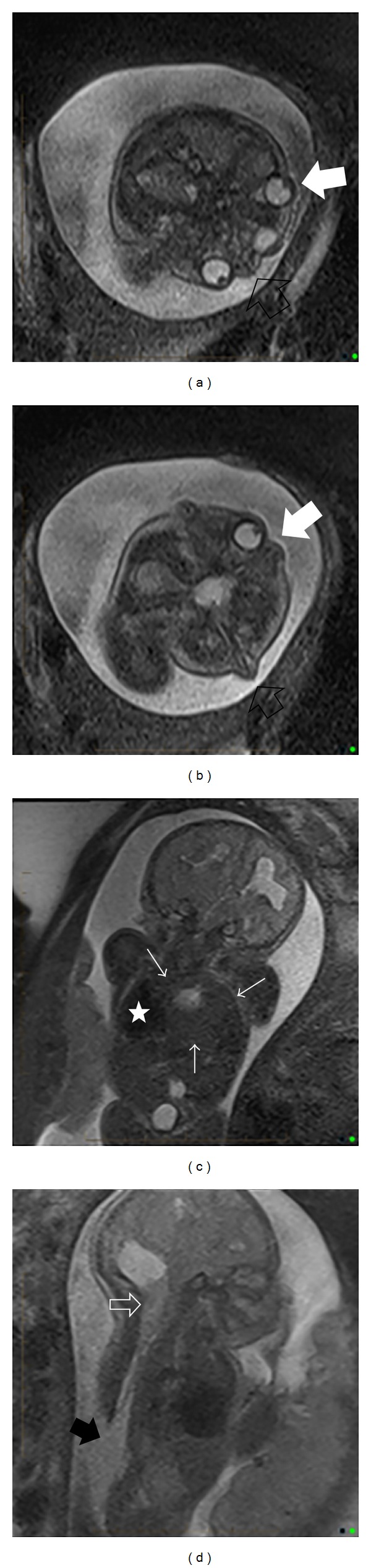
Axial images from fetal MRI (a and b) showing the left-sided (open black arrows) and right-sided (solid white arrows) fetal faces. Coronal image from fetal MRI (c) reveals a left-sided diaphragmatic hernia (thin white arrows) containing liver and bowel with displacement of the fetal heart (white star) to the right. Chiari II malformation with tonsillar herniation through the posterior foramen magnum (open white arrow) and associated lumbosacral spinal dysraphism (solid black arrow) is noted on a sagittal image (d).

A 29-year-old gravida 2 para 1 Caucasian female was referred for maternal-fetal-medicine consultation at 26-week gestation due to suspected fetal anomalies. Obstetrical ultrasound examination, confirmed by fetal magnetic resonance imaging (MRI), demonstrated craniofacial duplication, several abnormalities of the brain and skull, thoracolumbosacral dysraphism with neural tube defect and likely Chiari II malformation, large congenital diaphragmatic hernia (CDH) with liver and bowel noted in the left chest, hypoplastic left lung, and possible horseshoe kidney (Figure 1). Fetal echocardiogram findings were consistent with Tetralogy of Fallot (TOF). The parents had a multidisciplinary consultation with maternal-fetal medicine, neonatology, genetics, cardiology, radiology, and palliative care. Given the multiple, severe congenital anomalies, the medical team and family planned for limited resuscitative efforts and anticipated comfort care after birth.

A 2440 g male infant was delivered via repeat cesarean section at 36-week gestation secondary to preterm labor. At birth, the infant was pale and cyanotic with no spontaneous cry and poor tone. He had shallow spontaneous respirations with poor aeration on auscultation. The left mouth was small and fixed. No glottis was visualized when the laryngoscope was inserted into the right mouth. The infant was then wrapped in a warm blanket and given to the family to hold. He died in his parents' arms a few minutes after birth.

The parents consented to a complete postmortem evaluation. Physical exam demonstrated complete facial duplication with tetrophthalmos (four eyes), two noses, two mouths, two chins, and one fully formed ear on each side of the head with a hypoplastic pinna in the midline. Two anterior and two posterior fontanels were palpable. There was also a ruptured myelomeningocele measuring 6.3 cm × 3.9 cm in the thoracolumbar region and a scaphoid abdomen. The first and second toes of each foot overlapped and the nails were hypoplastic.

**Figure 2:**
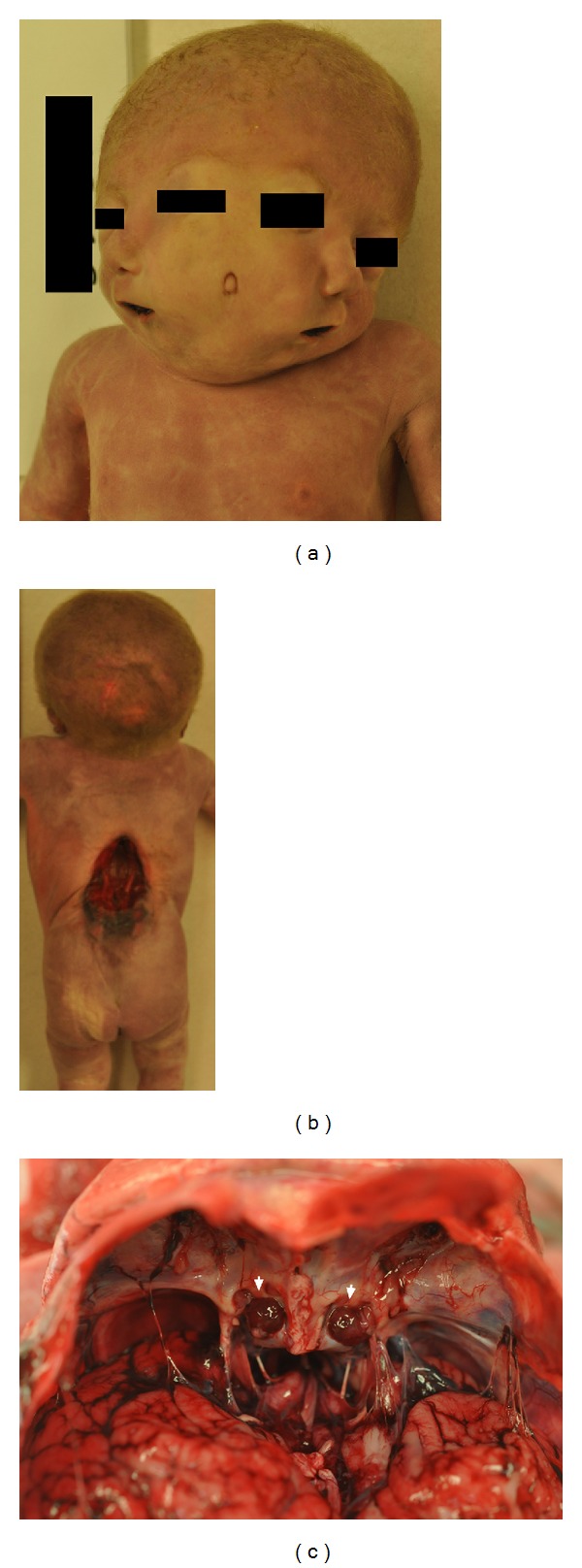
(a) Facial duplication with a hypoplastic pinna in the midline. (b) Open thoracolumbar myelomeningocele measuring 6.3 cm × 3.9 cm. (c) Anterior cranial fossa with two pituitary glands (white arrows).

Dissection of the cranial cavity revealed brain duplication with fusion of the parietal and occipital lobes of each brain. The right and left brains fused at the mesencephalon and brainstem. Each brain had an optic chiasm, a pituitary gland, and a single large ventricle with loss of the ependymal lining and no third ventricle identified. There was a single midbrain, rudimentary cerebellar tissue and fourth ventricle, atresia of the cerebral aqueduct with rudimentary and hypoplastic cerebral peduncles, bilateral absence of the corpus callosum, bilateral polymicrogyria, and dysplasia of the right and left cortices. Dissection of the thorax and abdomen revealed left CDH with intestine, stomach, spleen, and the left lobe of the liver herniated into the left pleural cavity with concomitant pulmonary hypoplasia. Cardiac findings included TOF (pulmonary atresia, ventricular septal defect, overriding aorta, and pulmonary arteries), atrial septal defect, patent ductus arteriosus, superior pulmonary veins draining to the left atrium (no inferior pulmonary veins), dilated right atrium, and hypoplastic left atrium. A common oropharynx connected two separate nasopharynxes and two separate oral cavities. There was a single larynx and esophagus. The intestines were malrotated with the appendix located in the left upper abdominal quadrant. Ectopic right and left kidneys were located in the lower abdomen and an accessory spleen was present. Testes were undescended bilaterally and the right testis was atrophic. The placental pathology revealed normal fetal membranes and chorionic villi consistent with third trimester gestation with a two-vessel umbilical cord (Figure 2).

Whole-genome microarray-based comparative genomic hybridization (aCGH) using Oxford Gene Technology revealed a male karyotype (46 X,Y), a 983 kb deletion on chromosome 4q34.3, a 562 kb gain on chromosome Xp22.31p22.2, and a 32 kb gain on chromosome 13q12.11. Quantitative polymerase chain reaction of maternal blood indicated the duplications on chromosomes Xp22.31p22.2 and 13q12.11 were maternally inherited. The 4q34.3 deletion was not maternally inherited, however. Unfortunately, due to the family's financial burden, genetic testing was not completed on the father.

## 3. Discussion

Diprosopus or craniofacial duplication is the rarest form of conjoined twinning, with an incidence of approximately 0.4% of all types of conjoined twins [1]. Historically, two main theoretical embryologic explanations have been considered: either a “fusion” of two parallel notochords in close proximity occurs or a “fission” of a single notochord occurs during the first few weeks after conception [2]. More recent theories include duplication of neural crest cell derivatives and mutations of the* Dix* homeobox gene [3, 4]. A spectrum of diprosopus exists from a duplication of only the nose to complete facial duplication similar to that of our patient [5]. Many other congenital anomalies have been reported to occur in conjunction with diprosopus, including various neurologic, cardiac, pulmonary, skeletal, and gastrointestinal system defects [5–8]. Conjoined twins have a high mortality, especially if they have other major congenital anomalies [8, 9].

There have been no previous reports of genetic mutations associated with diprosopus. The clinical significance of the maternally inherited duplications at Xp22.31p22.2 and 13q12.11 is unknown. There is a theoretical possibility of an X-linked recessive inheritance pattern since the X chromosome duplication has caused no disease or malformation in the mother, but this is unlikely as no reports of familial recurrence have previously been reported in the literature. The 4q34.3 deletion has been associated with cardiovascular abnormalities such as TOF [10, 11], but none of these genes are located in the deletion region in our case. Given the rare incidence of this disease and our inability to complete genetic testing of both parents, we are unable to conclude that any of the genetic findings are associated with our patient's congenital malformations and the family was counseled accordingly. However, a lack of genetic association for diprosopus continues to support an embryologic theory of abnormal twinning.

It should be noted that the prenatal diagnosis at 26 weeks somewhat limited perinatal management options. However, the combination of detailed ultrasound and MRI imaging provided accurate prenatal diagnoses so that the family and medical providers were able to develop a comprehensive plan of care in advance of the delivery. Almost three months before the birth, medical providers compassionately informed the family that a successful resuscitation was unlikely. The parents asked that providers to make an attempt to resuscitate, but if unsuccessful, they wanted to hold and baptize their son. During follow-up counseling, the family stated that the birth and death experiences were more peaceful than anticipated. They felt that the medical, emotional, and spiritual support provided by the multidisciplinary team significantly reduced their anxiety, facilitated their decision-making, and, ultimately, aided them in coping with their loss.

## References

[1] Edmonds LD, Layde PM (1982). Conjoined twins in the United States, 1970–1977. *Teratology*.

[2] Spencer R (1992). Conjoined twins: theoretical embryologic basis. *Teratology*.

[3] Carles D, Weichhold W, Alberti EM, Leger F, Pigeau F, Horovitz J (1995). Diprosopia revisited in light of the recognized role of neural crest cells in facial development. *Journal of Craniofacial Genetics and Developmental Biology*.

[4] Depew MJ, Lufkin T, Rubenstein JLR (2002). Specification of jaw subdivisions by *Dlx* genes. *Science*.

[5] Hähnel S, Schramm P, Hassfeld S, Steiner HH, Seitz A (2003). Craniofacial duplication (diprosopus): CT, MR imaging, and MR angiography findings—case report. *Radiology*.

[6] D’Armiento M, Falleti J, Maria Maruotti G, Martinelli P (2010). Diprosopus conjoined twins: radiologic, autoptic, and histologic study of a case. *Fetal and Pediatric Pathology*.

[7] Laor T, Stanek J, Leach JL (2012). Diprosopus tetraophthalmus: CT as a complement to autopsy. *The British Journal of Radiology*.

[8] Fernandes GL, Matsubara FK, Marques FK (2010). Three-dimensional prenatal diagnosis of monocephalus diprosopus tetraophthalmos. *Journal of Ultrasound in Medicine*.

[9] Spitz L, Kiely EM (2003). Conjoined Twins. *Journal of the American Medical Association*.

[10] Xu W, Ahmad A, Dagenais S, Iyer RK, Innis JW (2012). Chromosome 4q deletion syndrome: Narrowing the cardiovascular critical region to 4q32.2-q34.3. *The American Journal of Medical Genetics A*.

[11] Huang T, Lin AE, Cox GF (2002). Cardiac phenotypes in chromosome 4q- syndrome with and without a deletion of the dHAND gene. *Genetics in Medicine*.

